# Adherence challenges to therapeutic footwear among high risk diabetic foot populations in urban Southwest China

**DOI:** 10.1038/s41598-025-06323-z

**Published:** 2025-07-01

**Authors:** Wangqiao Zhu, Liuxue Lu, Lifang Huang, Sulan Long, Lina Ou, Meng Yang, Yongxian Wei, Yanfang Lu, Yunjiao Tang, Jia Liu

**Affiliations:** 1 Faculty of Nursing, Youjiang Medical University for Nationalities, Chengxiang Rd., Youjiang Disctrict, Baise, 533000 China; 2 Affiliated Hospital of Youjiang Medical University for Nationalities, Zhongshan Rd., Youjiang Disctrict, Baise, 533000 China; 3 Faculty of nursing, Nanchang Medical College, Nanchang, 330052 Jiangxi Province China; 4 Baise People’s Hospital, Chengxiang Rd., Youjiang Disctrict, Baise, 533000 China

**Keywords:** Diabetes, Information, Therapeutic footwear, Urban, China, Disease prevention, Health services, Diabetes complications, Patient education

## Abstract

Diabetic foot ulcers [DFUs] are severe complications of diabetes, and therapeutic footwear plays a crucial role in their prevention. However the factors influencing adherence to therapeutic among high-risk individuals in urban areas remain poorly understood. With increasing urbanization and migrant workers, this study aims to explore the challenges of adherence to therapeutic footwear among high-risk diabetic foot patients in urban area. This study employed a qualitative descriptive design. Data were collected through semi-structured interviews and focus group discussions, and analyzed using a thematic approach. A total of 25 participants (20 patients and 5 healthcare providers) were recruited through purposive sampling. Two key challenges of footwear adherence were identified: (1) inadequacy of consistent and reliable health resources, and (2) appearance-related barriers to footwear adherence: professionalism, fashion, and social perception. Participants reported inconsistent and unreliable health information, including confusion from online sources and varied hospital guidance, which reduced their confidence in wearing therapeutic footwear. Additionally, appearance-related barriers—such as workplace dress codes, fashion preferences, and fear of social judgment—discouraged use of therapeutic footwear in both professional and social settings. This study highlights the need to integrate social and functional elements into the design of therapeutic footwear. Nursing professionals play a critical role in enhancing communication and coordination around foot care. Enhancing access to reliable health information is essential for promoting DFU prevention, particularly among urban migrant populations.

## Introduction

Globally, the incidence of type 2 diabetes mellitus [T2DM] is projected to rise from 415 million in 2015 to 642 million by 2040^1^. In 2021, the countries with the largest numbers of adults with diabetes aged 20–79 were China (140.9 millions), India (74.2 millions) and Pakistan (33.0 millions). In China, this number is expected to grow from 116.4 million in 2019 to 147.2 million by 2045^2,3^. T2DM may present long-term complications, including cardiovascular disease, diabetic kidney disease, and diabetic foot ulcers [DFUs]^4,5^. The global prevalence of diabetic foot complications varies from 3% in Oceania to 13% in North America. On a worldwide scale, the average prevalence of DFUs is 6.4%, and approximately 1% of individuals with diabetes undergo lower-limb amputation^6^. Economically, DFUs impose a substantial burden: healthcare costs are 5.4 times higher in the year of the first ulcer episode and 2.6 times higher in subsequent occurrences^2^. In China, one study reported an annual DFU incidence of 8.1% and a recurrence rate of 31.6% within the first year. The amputation rate among DFU patients was recorded at 5.1%^7^. Specifically, in Baise City, local hospital data showed an increase in DFU incidence from 10.1% in 2019 to 14.6% in 2022 (with permission).

The World Health Organization [WHO] emphasizes self-care as a cornerstone of early diabetes detection and complication prevention^3^. Following the International Working Group on the Diabetic Foot [IWGDF], appropriate therapeutic footwear is essential for ulcer prevention, particularly for individuals at high-risk^8^. The WHO global diabetes guidelines also emphasize the importance of footwear education and regular foot examinations to monitor for neuropathy, impaired blood flow, and skin changes^3^. Health information resources and digital content platform play a pivotal role in preventing DFUs and avoiding limb amputations^1^. While prior studies have explored challenges to health information access, such as reliance on spiritual powers^9,10^. However, amid the continuous growth of cities and shifting population patterns, limited attention has been dedicated exclusively to studying these issues among high-risk urban populations. Many educational interventions lack effectiveness in establishing sustainable knowledge-sharing platforms^11,12^. Presently, adherence to foot self-care practice, including routine screening, avoiding barefoot walking, and wearing therapeutic footwear, remains insufficient^13,14^. Challenges to therapeutic footwear adherence, includes prioritizing practical functionality^15^ and a preference for the aesthetic aspects of therapeutic footwear^16^have been examined. Most therapeutic footwear designs prioritize function with limited attention to psychosocial or cultural factors^17^. For example, one study on custom-made footwear for foot ulcer recurrence stressed the importance of mechanical protection against external pressure during increased physical activity^18^.

Behavior change is determined by multiple interrelated factors, such as physical health, social and cultural environments, and personal attributes^19^. For instance, populations in South Asian face challenges in adapting to health behaviors relevant to diabetes management, including increased consumption of fats and sugars, sedentary lifestyles, and societal stigmas associated with the disease^20^. In China, sustained efforts toward gender equality have resulted in participation of women in higher education and white-collar professions, particularly in urban areas^21^. While this shift has alleviated the burden of heavy physical labor for many urban women, it has also presented new adaptation challenges. A study in the United States suggests that female sales personnel are often encouraged to adopt formal attire, including high-heeled shoes, to attract customers and receive positive evaluations^22^. Baise City, characterized by its mountainous terrain and rural landscapes, experiences a contrast in medical development between urban health centers and township health units. Urban areas, with increased resources, face heightened demands for superior healthcare amidst significant urbanization, economic growth, and an influx of rural-to-urban migrant workers.

Aligned with the 14th Five-Year Plan for National Informatization, the State Council of China emphasizes the advancement of extensive medical and health data integration. This initiative aims to consolidate existing resources, facilitating the open integration of medical data across departments, regions, and industries to foster collaborative construction and sharing^23^. Concurrently, the 2022 Law of the People’s Republic of China on the Protection of Women’s Rights and Interests underscores the nation’s commitment to promoting women’s involvement in economic, social, and political spheres, aligning with broader efforts to advance gender equality^24^. Human behavior, shaped by personal beliefs and social environmental elements, finds broad applications in psychology, education, and communication research. In the context of diabetic foot care, personal attributes and social interactions are likely to influence adherence to preventive measure. This study aims to explore the challenges associated with adherence to therapeutic footwear among high-risk populations for DFUs in urban areas.

## Method

### Design and setting

The study employed a qualitative descriptive design, utilizing both semi-structured interviews and focus group discussions for data collection. This approach facilitated a in-depth exploration of real-world issues, as perceived through the lens of participants’ experiences^25^. The straightforward nature of this method allowed researchers to understand participants’ experiences and perspectives, aligning with naturalistic inquiry principles that are essential for improving nursing practice and patient outcomes^26^. Interviews and focus group discussions were conducted at two local diabetic practicum bases affiliated with a local tertiary hospital that provides educational activities for communities. Baise City, located in the Guangxi Zhuang Autonomous Region of Southwest China, features a unique culture, social dynamics, and dialect due to its remote geography and ethnic diversity, which may influence local patients’ health behaviors.

### Participants

A total of 25 participants who visited the diabetic practicum bases for experience-sharing activities were recruited. Fifteen individuals with diabetes participated in semi-structured interviews. Additionally, two focus groups were conducted: the first included five individuals with diabetes, and the second comprised five healthcare providers, including nurses, physicians, and podiatrists. The inclusion criteria for individuals with diabetes were: (1) a confirmed diagnosis of T2DM, (2) identified as a DFU high-risk group according to IWGDF 2023 criteria (a history of foot ulcer, a lower-extremity amputation (minor or major), or end-stage renal disease^6^(3) received recommendation of diabetic footwear from healthcare providers (4) age 18 years or older Mini-Mental State Examination (MMSE score of 24 or higher for cognitive function in individuals aged 60 years or older), (5) have resided in the urban area for 6 months or more, (6) proficient in Chinese, (7) willingness to participate. Healthcare providers were eligible if they had at least one year of experience in diabetic foot care, were involved in therapeutic footwear management, and worked in diabetes-related healthcare settings. Purposive sampling was employed to intentionally identify participants with diverse age groups, ethnic backgrounds, genders, and disease duration. The first participant was referred by a clinical healthcare provider, with subsequent participants recruited through snowball sampling^27^. The written informed consent inform was obtained from all participants. This study follows the Consolidated Criteria for Reporting Qualitative Research (COREQ): a 32-item checklist.

### Data collection

Data were collected between May and September 2024 through semi-structured interviews and focus group discussions. The interview team included one main researcher (WQZ) and one assistant (JL). Both were knowledgeable about diabetes foot care and had participated in a role-play stimulation training course before data collection. Prior to interviews, consent was obtained from the managers of the diabetic practicum bases. Participants were invited to a quiet, uninterrupted room to ensure comfort and minimal interruptions. Following a brief introduction to the study’s objectives, procedures, and the details of therapeutic footwear, informed consent was then acquired. An interview guide (Table 1), crafted with reference to literature reviews^2,5^underwent content validity assessment by six experts, demonstrating a validity score of 0.92^28^. Open-ended questions were posed to understand the perspectives of participants, accompanied by auxiliary questions such as “how do you find out about ways to prevent foot ulcers?” and “what things do you think affect your choice of special shoes for foot care?” Interviews lasted 45–60 min, were audio-recorded and supplemented with field notes. The saturation of data was achieved until no new information emerged^27^. All records were securely maintained to ensure the participants privacy and confidentiality. Participants were genuinely acknowledged for their contributions.

**Table 1:** Interview guide.

What are the challenges of adherence to therapeutic footwear?
● How do you think about therapeutic footwear?
● Do you feel difficult to wear therapeutic footwear instead of general shoe?
● Why couldn’t you insist on wearing it?
● Are there any other reasons why you could not wear it regularly?

Focus groups were conducted in a similar setting, facilitated by the main researcher with assistance from JL. The interview guide was adapted to foster interactive discussion, with prompts encouraging the exploration of shared and differing viewpoints. Each session lasted approximately 60–75 min and was audio-recorded with consent. Field notes captured group dynamics and non-verbal cues. These sessions provided additional insight into footwear adherence from both patient and provider perspectives.

### Data analysis

Audio recordings were analyzed through a six-phrase thematic analysis strategy: transcribing, coding, sub-themes combination, themes review, themes identification, and writing up^29^. Firstly, audio records were transcribed verbatim and cross-referenced with field notes. Then, data analysis was conducted inductively and deductively. Transcribes were coded independently, comparing it with research questions line by line. Thirdly, sub-themes were formed through analyzing coded data that had a similar explanation to research questions. For example, “some of them will mention a little about foot care” and “different physicians say differently” were categorized under the sub-theme “inconsistent education from hospitals”. Fourthly, sub-themes were reviewed for differences and similarities. Fifthly, related sub-themes were combined into overarching themes. Finally, the findings were then synthesized into a written narrative supported by direct participant quotations. Any discrepancies during the analysis process were discussed and re-checked among the research team. Participants were also consulted to validate the accuracy of the identified interview categories in representing their thoughts and perspectives authentically. All data were systematically tracked and managed utilizing NVivo 12 (QSR International Pty Ltd). In this paper, participant categories are abbreviated as follows: P for persons with diabetes, N for nurse, PH for physician, and PO for podiatrist.

### Trustworthiness

Drawing upon the criteria outlined by Lincoln and Guba, trustworthiness in this qualitative inquiry was diligently ensured^27^. Employing a member-checking approach to bolster credibility, the obtained data underwent meticulous summarization, with participants actively participating in the review process. Dependability was assured through rigorous auditing of all research processes and documents. To enhance confirmability, at least two researchers conducted a thorough re-examination and review of the data, addressing any discrepancies. To mitigate researcher bias, a reflective journal was consistently maintained after each interview. Additionally, a detailed and comprehensive depiction of the findings was presented to facilitate the transferability of the study.

### Ethics approval

This study was conducted in accordance with the Declaration of Helsinki and received approval from the institutional review board of the Chinese health administration agency in the local district (1224/2024).

## Results

### Participants characteristics

A total of 20 patients were interviewed. The average age of participants was 50.8 ranged from 47 to 66 years old. Over half of the participants were males (*n* = 12; 60%), with Zhuang ethnicity (*n* = 12; 60%) and urban residence (*n* = 11; 55%) being predominant. The majority of participants had senior high school education (*n* = 13; 65%). For income, most participants were in the range of 4001 ~ 5000 CNY (*n* = 12; 60%). Most participants had one time of ulcer onset (*n* = 12; 60%) and exhibited a disease duration of 5 years or above (*n* = 10; 50%). Nearly one third of the participants were retired (*n* = 6; 30%). A detailed characteristic of participants was in Table 2.

**Table 2:** Characteristics of patients (*n* = 20).

Characteristics	Group	*N*	%
Age (years)	< 50	3	15
	51–60	9	45
	> 61	8	40
Gender	Female	8	40
	Male	12	60
Residence	Urban	11	55
	Rural	9	45
Ethnicity	Han	6	30
	Zhuang	12	60
	Yao	2	10
Highest level of education	Junior high school or lower	5	25
	Senior high school	13	65
	College or above	2	10
Duration of disease (years)	1–2	3	15
	3–4	7	35
	5 or above	10	50
Occupation	Security guard	4	20
	Gardener	2	10
	Construction worker	3	15
	Bricklayer	2	10
	Governmental employee	3	15
	Retirement	6	30
Monthly Income in CNY (1 US dollar = 7.16 CNY)	4000 or lower	5	25
	4001 ~ 5000	12	60
	5001 ~ 6000	2	10
	6001 or more	1	5
Previous foot ulcer	One time	12	60
	Two times or above	8	40

**Table 3:** Characteristics of healthcare providers (*n* = 5).

Characteristics	Group	*N*	%
Age (years)	≤ 50	3	60
	> 50	2	40
Gender	Female	3	60
	Male	2	40
Residence	Urban	4	80
	Rural	1	20
Ethnicity	Han	2	40
	Zhuang	3	60
Highest level of education	Senior high school	1	20
	College or above	4	80
Occupation	Nurse	3	60
	Physician	1	20
	Podiatrist	1	20
Monthly Income in CNY (1 US dollar = 7.16 CNY)	5000 or lower	1	20
	5001 ~ 6000	3	60
	6001 or more	1	20

A total of 5 healthcare providers participated in a focus group discussion. The majority were under 50 years old (*n* = 3; 60%), and most were female (*n* = 3; 60%). Most participants resided in urban areas (*n* = 4; 80%) and identified as Zhuang ethnicity (*n* = 3; 60%). The majority had attained a college-level education or above (*n* = 4; 80%). Occupational roles included nurses (*n* = 3; 60%), one physician (*n* = 1; 20%), and one podiatrist (*n* = 1; 20%). In terms of monthly income, most providers reported earning between 5001 and 6000 CNY (*n* = 3; 60%). Detailed characteristics of the healthcare provider participants are shown in Table 3.

### Overview of themes

This qualitative study identified two primary themes reflecting the challenges faced by high-risk urban populations in adhering to therapeutic footwear for DFU prevention: (1) inadequacy of consistent and reliable resources and (2) appearance-related barriers to footwear adherence: Professionalism, fashion, and social perception.

### Theme 1: inadequacy of consistent and reliable health resources

Urban residents often have access to diverse health information sources, encompassing interactions with healthcare providers such as nurses or physicians, perusing printed materials, or engaging with modern technological platforms. However, challenges emerged around the reliability and consistency of this information, which impacted their adherence to therapeutic footwear. These challenges can be distilled into two sub-themes as follows: (1) uncertainty on health information (2) inconsistent education from hospital.

### Uncertainty on health information online

One participant conveyed a strong disapproval of depending on supernatural beliefs or powers for matters related to disease and health. Instead, this individual exhibited a proactive approach towards seeking health advice to prevent DFUs, often turning to the internet as a valuable resource. However, there were moments of doubt regarding the authenticity and reliability of the health information found online. This uncertainty emerged as a barrier to adherence.

“I don’t believe in buddle, I don’t think it works. When I have time, I will turn on my cell phone and search short video online; en…however, you know nowadays the Internet is very complex. Sometimes there are fake information and I get confused what I can really listen to.” (P 2).

While healthcare providers served as the primary reservoir of reliable information, numerous alternative channels existed for acquiring health-related information. These channels included educational booklets distributed during hospitalization, public newspapers, television programs, and more. These resources proved highly advantageous in enhancing awareness and advocating for foot protection strategies. Nevertheless, concerns began to emerge regarding the quality of television-based educational programs. When juxtaposed with previous experiences, such as online shopping, concerns about the accuracy of these materials also hindered patients’ ability to trust and follow the advice they received.

“During hospitalization, healthcare providers teach us how to check foot, not to walk in barefoot, and so on. I also learn from the health education manual and from the health billboard along the hospital corridor. Additionally, you can learn from newspapers, TV programs. However, with TV programs, you sometimes unsure about the accurancy of what they say. Similarly, some advertisements on the Internet promote their products, but they often prioritize profit over accuracy.” (P 4).

“Sometimes those articles or videos look like health advice, but in the end they just want to sell something. You really have to be careful.” (FGD-P16).

### Inconsistent education from hospitals

A significant barrier to adherence was the inconsistency in health education across healthcare providers. Despite the ready availability of medical care within the hospital setting, patients often found themselves in perplexing situations due to the marked inconsistency in the health education provided by various physicians. This inconsistency in healthcare advice caused confusion and undermined their ability to adopt consistent footwear practices.

“Different physicians say differently. I am not sure who should I listen to. Sometimes some physicians don’t teach anything. Actually my home is not very far from hospital. It is very convenient. I almost have been to them (hospitals) all because my diabetes problem has been a long time. Many physicians and nurses know me.” (P 10).

A participant who had experienced medical treatment across multiple hospitals generously shared their valuable perspective. They observed a striking inconsistency in the emphasis placed on knowledge about foot self-care for individuals at risk of DFUs, particularly when compared to the consistent stress on glycemic testing and medication adherence as essential components of the medical regimen.

“I went to see physicians in many places. For foot care, I really don’t know too much about it. Some of them will mention a little about foot care, but some don’t. Blood glucose test is always stressed, and also medication taking.” (P 15).

### Theme 2: Appearance-related barriers to footwear adherence: professionalism, fashion, and social perception

While participants generally acknowledged the medical advantages of therapeutic footwear in preventing DFUs, many struggled to consistently wear them due to concerns about how the footwear fit within their work attire, social expectations, and personal sense of style. These concerns created a tension between medical adherence and maintaining a socially and professionally acceptable appearance. Two sub-themes emerged: (1) Workplace dress codes and expectations (2) Aesthetic and social perceptions of therapeutic footwear.

### Workplace dress codes and expectations

Participants working in formal or public-facing environments found therapeutic footwear incompatible with their workplace dress codes. A female participant working as a secretary described how her role required not only formal attire like suits and leather shoes but also maintaining a polished, professional appearance during after-hours work functions. For her, the therapeutic footwear visually conflicted with expectations of office-appropriate style.

“Besides from normal paper work in office, there are many receptions after work, for example having dinner and other communications…As a secretary, I cannot wear this (footwear) in my office. I need to wear suit and leather shoe. And that’s how it looks formal.” (P 8).

In a different context, a male security guard emphasized how public visibility influenced his clothing choices. Although he acknowledged the health importance of the footwear, he believed wearing it would undermine his professional image and credibility in front of office staff and superiors.

“As a security guard here at the corporate office, I see a lot of the staff and managers coming through the gate every day. I have to wear a formal uniform and nice leather shoes, so I can’t wear those therapeutic shoes. Honestly, I think if I showed up in them, people would question my professionalism. It’s all about making a good impression, you know?” (P 12).

“At work, we’re expected to look neat and professional. These shoes just don’t match the work. If I wear these shoes, people might not take me seriously.” (FGD-P18).

### Aesthetic and social perceptions of therapeutic footwear

Participants, especially women, expressed dissatisfaction with the visual design of therapeutic footwear, often describing it as “elderly” or unattractive. One female office worker emphasized how such footwear conflicted with her sense of style and femininity, especially in social settings. Her concern extended to peer judgment and the emotional discomfort of not fitting in.

“I don’t like this shoe (therapeutic footwear). It is like elderly shoe. And on weekends, I will go dancing with friends in the park. I cannot wear this.” “It is even less likely to wear this in work. If I wear this in office, wouldn’t my colleagues laugh at me?… normally I wear kitten heels or Stilettos when I work.”(P 3).

Other participants echoed similar discomfort, but focused more on social norms and expectations. One participant emphasized that wearing therapeutic footwear in her office would feel “strange” and nonconforming.

“I cannot wear this footwear to work… I cannot wear this in my office, no body wears like this. It looks strange. Normally I am requested to wear suit in work.” (P 11).

“If I wore these shoes to work, I think people would find it strange. No one else wears them, and they just don’t fit the usual look.” (FGD-P19).

### Perspectives from the healthcare provider

A nurse corroborated patients’ concerns regarding the reliability of online and media-based health information. She noted that many patients frequently encountered misleading or promotional content, which complicated their understanding of appropriate diabetic foot care.

“I see patients come in with all sorts of ideas they’ve picked up from TV or the internet. Some of it’s helpful, but a lot is misleading. I always tell them to double-check with us before trusting what they see, especially ads that focus more on selling than informing.” (FGD-N 2).

A physician emphasized the inconsistency in foot care education across clinical settings, echoing patients’ frustration with receiving conflicting or insufficient guidance about therapeutic footwear. He acknowledged the systemic need for greater alignment in patient education.

“To be honest, the focus on blood sugar and medication is pretty consistent, but foot care advice can really vary between hospitals. Some places make it a priority, while others barely touch on it. We need to be more aligned on this.” (FGD-PH 1).

In line with patients’ dissatisfaction with the aesthetic aspects of therapeutic footwear, a podiatrist observed that appearance-related concerns significantly influence patients’ willingness to wear the shoes. These concerns were often tied to perceived social stigma or reduced participation in social activities.

“I’ve heard some patients say they don’t want to wear therapeutic shoes because they look old-fashioned. They feel like they can’t dance or dress well in them, and they worry about what coworkers might think.” (FGD-PO 1).

## Discussion

This study provided insights into the challenges of adherence to therapeutic footwear among individuals at risk of DFUs, focusing on the difficulties they face in an increasingly urbanized and informatized environment. Compared with people living in relatively less-developed rural areas, many participants experienced improvements in accessing health information and medical care in hospitals, as well as having time available for individual foot care. However, new challenges to adherence emerged due to changing individual needs along with transformations in social, environmental and economic conditions within urban areas. These findings suggest that urbanization presents both facilitators and barriers to adherence, requiring tailored strategies to address its unique challenges.

### Reliability challenges in health information

A tendency for acquiring health information online was found among people at risk of DFUs in urban areas. This increased access to information can help raise awareness of diabetic foot self-care. This finding was consistent with one Canadian study revealing that participants realized the necessity of engaging in self-directed learning and attempted to Google searching on treatment options, medications and strategies of foot self-care. It helped to decrease the frequency of hospital treatments^30^. Nowadays, the Internet has become the main source of information for patients with chronic diseases^31^particularly among those with higher education backgrounds^32^. However, this finding contrasts with studies conducted in regions where cultural beliefs significantly influence health practices. For example, in Saudi Arabia, the state of health and relevant health practices were often perceived as predetermined by a higher power^33^. People held the perspective that they had to accept their fate on health and sought strength and guidance from deities to aid in foot ulcer recovery^34^. With the advent of the information age, people residing in urban areas had more opportunities to access health information than their rural counterparts^35^. Despite the preference for self-searching from contemporary channels being beneficial for enhancing knowledge about foot care to some degree, another challenge reflected in our study was encountering fake or commercially driven messages. It plays an important role in influencing adherence to therapeutic footwear, yet it was not noticed formerly. By comparison, one previous study about pediatric diabetes care revealed that parents perceived difficulty to assess reliable source of information about treatment and prognosis of diabetes online^8^. One study on general diabetes education showed indeterminacy regarding website content without local-language instruction among patients with T2DM^10^. Anxiety and concern about the reliability of the increasing abundant propaganda on the Internet were perceived by young parents caring for children with type 1 diabetes^36^. This finding highlights the need to address the patients’ desire for independent learning and offer credible resources for informed decision-making.

### Inconsistent education in healthcare

In urban districts, access to hospital-based foot care services is perceived instant compared to rural areas. For instance, a U.S. study showed that significantly more urban counties had access to the National Diabetes Prevention Program^37^. The utilization rates of medical resources and consultations with physicians were dramatically higher in urban areas among patients with diabetes^38^. Contrastingly, studies from other countries reveal challenges faced by patients in rural areas. In Ireland, patients with diabetic foot disease in rural regions sometimes opted to wait and monitor the progression of foot problems before seeking medical help, primarily due to the long distances to hospitals^39^. An Australian study also reported that patients in rural districts experience difficulties in making physicians appointments^40^. In comparison with primary or township health centers, health resources were skewed more towards hospital. The disparity in the distribution of medical human resources between rural and urban regions remain significant^41^. Despite the enhanced accessibility of hospital-based foot care services in urban regions, a significant challenge identified in the present study is the inconsistency in foot care education among different healthcare providers. This inconsistency reduced patient’s confidence in recommended practices, thereby hindering adherence to therapeutic footwear^42^. A Jordanian study identified variations in the guidance on foot self-care provided by healthcare providers affiliated with different institutions in the country, which indicated variations in the quality of care offered^43^. Anders and Smith conducted a study focused on developing a resource for individuals with diabetes to prevent foot problems. The study revealed numerous inconsistencies in the health information materials provided by various health providers^42^. To address these challenges, it is suggested to construct a standardized information communication platform for diabetic foot care across healthcare facilities.

### Workplace barriers and professional image

In urban settings, some male participants in this study expressed a preference for leather shoes due to their social function, believing they enhanced personal impressions during social interactions. This reduced the willingness to use therapeutic footwear, increasing the risk of external pressure on the foot. For instance, a study on hospital attire showed that patients advocated for linen dressing instead of traditional hospital gowns, as it was both comfortable and preserved their dignity^44^. An article about medical uniforms also revealed that physicians wearing white coats were considered professional and trustworthy, building up a stronger patient-physician relationship^45^. Conversely, a certain study found that patients valued the practical functions of shoes over therapeutic benefits, for example slippers used in housework, though it was not recommended^16^. Therapeutic footwear was also perceived as unsuitable for outdoor working^15^. A UK study noted that prosthetics made it impossible for patients to return to their previous jobs due to mobility issues^46^. In the modern era, with the increasing economic prosperity in urban areas, more and more attention has been given to overall well-being, which is determined by broad social and environmental elements^47^. These findings highlight the need for newly designed therapeutic footwear that balance social appeal and therapeutic benefits simultaneously^48^.

### Fashion, femininity, and social stigma

Some female participants believed the appearance of therapeutic footwear negatively affected their femininity in the present study. They described the shoes as bulky or unattractive, which affected their self-image and social interactions^16^. The unfamiliar appearance impacted their sense of beauty, making them feel different on the street^49^. One study conducted in South West England found that some participants declined to try footwear because it did not match their preferred clothing styles and resembled men’s winter shoes^32^. Discriminatively, a study in Barbados found that opting for public healthcare still involved out of pocket expense, such as materials and therapeutic footwear, leading to poor adherence to therapeutic footwear use^50^. One UK article revealed that for participants in suburban districts, the primary concern for DFU prevention was the financial burden, as accumulated medical expenses worsened the situation. Economic hardship reduced motivation for seeking medical foot care^51^. In this study, some retired individuals in urban areas had more leisure time and a preference for pursuing overall well-being alongside therapeutic footwear usage. While, in rural areas, most women primarily undertook household duties throughout their lives, further limiting their efforts to take adequate foot care. A study on education for the prevention of foot ulcers demonstrated that female patients in rural counties were restricted in time for foot care education owing to extensive housework and caring for preschool children^52^. The way of life in which “men cultivate and women weave” has existed for a long time in many Asian countries. This phenomenon is still observed, especially in rural areas^20^. As time progresses and the government continues to promote gender equality in society, more women have gained equal rights in both education and employment. A growing number of women have secured jobs and enjoy high-quality benefits during retirement in urban districts^21^. Despite social status changes, which have freed the urban female population from the burden of heavy physical labor to some extent, high-heeled or dancing shoe were preferred over therapeutic footwear to accommodate particular work and entertainment environments. Although this was not known earlier, it heightened the potential risk of DFUs. Cultural activities, such as folk dance and traditional songs were widely favored by local people as way of recreation^53^. Additionally, newly emerging square dancing was remarkably popular among the general public as a post-work leisure activity^54^. Specialized dancing shoes were the main equipment for these activities, but they increased external pressure on the foot. In comparison, a previous study about commercial services demonstrated that formally dressed female sellers (e.g., in uniform, high-heeled shoes) were deemed to influence customer evaluation^22^. A flexible schedule for wearing therapeutic footwear, coordinating therapy, work and interests, is suggested, as well as the artistic consideration^16^.

### Strength and limitations

This study offers valuable insights into the challenges of adherence to therapeutic footwear. It considered about the impact of urbanization and the experiences of migrant workers. However, several limitations should be recognized. One limitation of this research is that the study was conducted within two diabetic practicum settings. This narrow focus may limit the generalizability of the findings, as the specific practices, resources, and patient demographics of this setting may not reflect those of other healthcare environments. This study gain perspectives from only five healthcare providers. Inclusion of diverse stakeholders, such as community staff members and volunteers, would enhance the breadth of understanding.

## Conclusion

The determinants of individual psychological needs and social interactions dynamically impacted adherence to therapeutic footwear among individuals at risk of DFUs. Social pressures, cultural perceptions, inconsistent foot care education, and unreliable health resources are significant barriers that impede adherence. Nurse specialists should promote socially adaptable footwear and patient-centered DFU education. Strengthening reliable health resources and improving communication across health units are essential for effective DFU prevention.

### Disclosure statement

The authors report no conflicts of interest.

## Data Availability

Data collected for this study are available from the corresponding author (JL) on reasonable request.
